# Does stereopsis account for the link between motor and social skills in adults?

**DOI:** 10.1186/s13229-018-0234-4

**Published:** 2018-10-24

**Authors:** Danielle Smith, Danielle Ropar, Harriet A Allen

**Affiliations:** 1 Research and Development Department, Cumbria Partnership NHS Foundation Trust, Carleton Clinic, Carlisle, CA1 3SX UK; 2 School of Psychology, University of Nottingham, Nottingham, NG7 2RD UK

**Keywords:** Stereopsis, Stereoability, Depth perception, Motor skills, Social skills, Factor analysis, Path analysis

## Abstract

**Background:**

Experimental and longitudinal evidence suggests that motor proficiency plays an important role in the development of social skills. However, stereopsis, or depth perception, may also play a fundamental role in social skill development either indirectly through its impact on motor skills or through a more direct route. To date, no systematic study has investigated the relationship between social skills and motor ability in the general adult population, and whether poor stereopsis may contribute to this association. This has implications for clinical populations since research has shown associations between motor abnormalities and social skills, as well as reduced depth perception in autism spectrum disorder and developmental coordination disorder.

**Methods:**

Six hundred fifty adults completed three validated questionnaires, the stereopsis screening inventory, the Adult Developmental Coordination Disorder Checklist, and the Autism Spectrum Quotient.

**Results:**

An exploratory factor analysis on pooled items across all measures revealed 10 factors that were largely composed of items from a single scale, indicating that any co-occurrence of poor stereopsis, reduced motor proficiency, and difficulties with social interaction cannot be attributed to a single underlying mechanism. Correlations between extracted factor scores found associations between motor skill and social skill.

**Conclusions:**

Mediation analyses suggested that whilst fine motor skill and coordination explained the relationship between stereopsis and social skill to some extent, stereopsis nonetheless exerted a substantial direct effect upon social skill. This is the first study to demonstrate that the functional significance of stereopsis is not limited to motor ability and may directly impact upon social functioning.

**Electronic supplementary material:**

The online version of this article (10.1186/s13229-018-0234-4) contains supplementary material, which is available to authorized users.

## Background

Motor ability exhibits rich and complex relationships with regards to other cognitive domains [1]. A basic movement repertoire of functional actions involving both fine (such as pointing a finger, eye movements) and gross (such as arm gestures, walking together) motor domains aids in the initiation and sustainment of successful social interactions [2]. For instance, motor control plays an important role in joint attention (e.g. head-turning, reaching, pointing) and imitation [3], both crucial components of social relations [4]. A relationship between social and motor abilities has been identified in typically developing children as young as 8 months [5], with development from crawling to walking encouraging the use of more advanced social behaviours, such as initiation of bids for joint attention and directed gestures [6, 7]. Other research, using longitudinal designs, reported relationships between motor function at 5–6 years and a range of social behaviours at 6–7 years [8], and between motor abilities at 6–7 years and social status with peers at 9–10 years [9]. Additionally, a reduction in social play and increased social reticence has been noted in children with poor motor skills [10].

Although there appears to be sufficient evidence to support a link between motor and social skills, our understanding of this relationship still has important gaps that need to be addressed. Firstly, although evidence for a relationship between motor and social skills has been demonstrated in several studies with typically developing children, it remains unclear whether this relationship would extend into early adulthood. Secondly, previous literature exploring the link between motor and social functioning has neglected important visual skills, such as depth perception, which may help contribute to the relationship between these two domains. For example, there are clear links between depth perception in terms of stereoacuity (the estimation of depth from combining information from two eyes) and the physical manipulation of objects, which may help explain poor coordination or clumsiness. More specifically, the shape of the hand is wider and less accurate when reaching and grasping, and the time taken for the reach is much slower in individuals where stereopsis is reduced or absent in adults [11–14], with even larger errors in these tasks for children with reduced stereoacuity [15–17]. Poor depth perception can also impact upon gross motor skills such as walking; adults with reduced stereoacuity also demonstrate differences in gait with a more cautious approach, higher toe clearance, and increased hesitation [18, 19]. All of these skills may have implications for the likelihood of taking part in team, motor, and social activities; however, this relationship has rarely been studied.

Depth perception and stereopsis may also impact on social skills more directly by influencing social behaviour, perhaps via social norms. When interacting with another individual, we need to determine and maintain an appropriate amount of personal space. Stereo depth cues are most useful in this peri-personal space [20] suggesting those with reduced ability to judge this might inadvertently violate these norms. Good stereo ability requires good alignment and vergence of the eyes, but there is also evidence that those with strabismus (i.e. poor alignment of the eyes) experience social exclusion [21]. There are also preliminary links between poorly regulated eye contact and social abilities [22]. Finally, Kuang et al. [23] found a relationship between stereopsis and quality of life in older people, but the mechanisms for this are, as yet, unclear. There are, therefore several ways in which depth perception might impact upon social skills either indirectly through motor skills, or through a more direct route. To date, however, research has only investigated the links between motor and social ability and the links between motor and visual abilities separately.

The potential relationship between motor, social, and visual abilities has important implications for understanding developmental disorders [24–26]. Poor social skills are central to the diagnostic criteria of ASD [27]. Gross and fine motor impairment as well as difficulties in motor planning have been reported in up to 90% of those with ASD [28–33]. Significant correlations between motor skills and socialisation [34] and degree of social impairment [35–37] have been found in children with ASD. Motor dysfunction is also central to the diagnosis of DCD (also referred to as ‘dyspraxia’ and affects around 5% of the population [31]), and there has been an increasing interest in the social functioning of individuals with DCD in recent years. Both clinical and screening studies have reported significant relationships between motor abilities and parent-reported peer or social problems [38–41], showing children with impaired motor skills engaging in more solitary-type activities and generally being more isolated from their peers. There is also evidence from parental report and empirical work that individuals with autism show violations of personal space with others which might impact upon social acceptance [42]. Finding a relationship between stereopsis, motor ability, and social ability might provide a useful avenue for understanding, and perhaps treating these developmental disorders.

From the few studies that have made a direct comparison between ASD and DCD, it would appear that both disorders exhibit a similar range of social and motor difficulties [43, 44]. Importantly, however, it may be that the co-occurrence of these impairments is attributable to another underlying factor. The majority of studies reporting stereoacuity in ASD indicate that those with ASD are less sensitive to binocular disparity than their TD counterparts or normative data [45–51]. There is one contradictory finding. Milne, Griffiths, Buckley, and Scope [52] used the Frisby stereotest and found no significant group difference in stereoacuity between the TD and ASD groups. However, Anketell et al. [46, 47] found differences using this stereotest. A general stereopsis deficit has also been observed in those with DCD; Creavin, Lingam, Northstone, and Williams [53] reported that those with DCD were on average 8 percentage points more likely to have impaired stereopsis (i.e. stereoacuity of higher than 60 arc s) than their TD peers (a 44.5% relative increase), and those with severe DCD were more likely to show evidence of poor depth perception than those with moderate DCD.

### The current study

Despite the suggestion from within clinical groups that poor depth perception, or stereopsis, may be linked to both motor skills and social abilities, there has been little research to investigate the relationship between these three abilities. Furthermore, previous work that has investigated the relationship between motor and social skills has focused on children, with a remarkable paucity of research involving adults. It is essential to initially establish the links between stereopsis, motor, and social skills in a larger general population where these skills vary before exploring these relationships in clinical populations, such as ASD, which are often complicated with other co-occurring conditions. Through measuring autistic traits in a large sample within the general population, this study will be able to identify any potential links, either direct or indirect, between depth perception and social skills. This work will help define more specific questions to explore this area further in ASD populations. This study has two primary aims. The first is to extend the previous research linking motor and social skill impairment in children to a typical adult sample, identifying the possible later consequences of early deficit in these domains. Secondly, to examine which particular aspects of motor and social skill impairment were contributed to by reduced stereopsis; that is, if the effects of poor stereoacuity are strong enough to be able to affect social skill either directly or through mediation by motor ability.

## Methods

### Participants and recruitment

Ethical permission from the University of Nottingham’s School of Psychology Ethics Committee was granted prior to recruitment. Participants were sampled opportunistically from Reddit (www.reddit.com; *n* = 311, 47.8%), social media and email (*n* = 193, 29.7%), and an internal recruitment system for undergraduate students at the University of Nottingham for partial completion of course credit (*n* = 146, 22.5%).

Potential participants were provided with a paragraph explaining the study and a hyper-link taking them to the survey website. Although all materials used were originally developed as ‘pen-and-paper’ questionnaires, it appears there is little variation in responses when questionnaires are presented on-line [54, 55]. Individuals were advised the completion of the study would take approximately 20 min. All participants were offered the chance to enter into a prize draw for one of two £15 vouchers.

The sample included 650 participants aged between 16 and 70 (mean 26.46 ± 10) years. Demographic data in the form of gender, age, and occupation were collected, though these were optional. There were 227 males (age 27.01 ± 10.31; range = 16–67 years), 369 females (age 26.24 ± 9.79; range = 16–70 years), and 6 who identified as “other” (age 25.33 ± 4.23; range = 21–33 years). The age values were not of a normal univariate distribution for any gender group; all groups had positively skewed age distributions, and the ages of the “male” and “other” genders were platykurtic. However, age distributions for each group were roughly equivalent according to a two-sample Kolmogorov-Smirnov test (male vs female *D* = 0.1, *p* = 0.08; male vs other *D* = 0.3, *p* = 0.6; female vs other *D* = 0.4, *p* = 0.2).

Of the participants who reported an occupation (89.5%, *n* = 582), 54.1% (*n* = 315) reported that they were enrolled in secondary or tertiary education, 38% (*n* = 221) were in employment, and 6.19% (*n* = 36) were not in work or education. More details regarding occupation, including a breakdown by industry, can be seen in Additional file 1: Table S1. Diagnoses were not collected as part of the demographical data. However, a number of participants (3.69%, *n* = 24) disclosed various psychiatric or organic illness via the feedback section of the questionnaire. 0.92% (*n* = 6) reported they had been diagnosed with an ASD (all self-described as Asperger’s syndrome), and 1.85% (*n* = 12) reported amblyopia, strabismus or general “poor vision”. All diagnostic disclosures are summarised in Additional file 2: Table S2.

### Materials

The questionnaires were presented in a fixed order, beginning with the Stereopsis Screening Inventory [56], then the autostereogram self-asssessment, followed by the Adult Developmental Disorder Checklist [57] and the Autism Spectrum Quotient [58]. Finally, demographics including age and gender were requested, but these were optional.

### Stereoacuity

The Stereopsis Screening Inventory (SSI) is a self-report screening inventory for stereopsis [56]. It is composed of 10 statements with 5 response options (never, seldom, occasionally, frequently, and always). Coren and Hakstian [56] demonstrated that the scores obtained using the SSI correlate highly (*r* = .8) with laboratory measures of stereopsis such as the TNO test, with others demonstrating a moderate relationship [59] between these measures (*r* = .34). Recommended cut-offs are 17 for moderate stereopsis deficit and 30 for major stereopsis deficit.

**Fig. 1:**
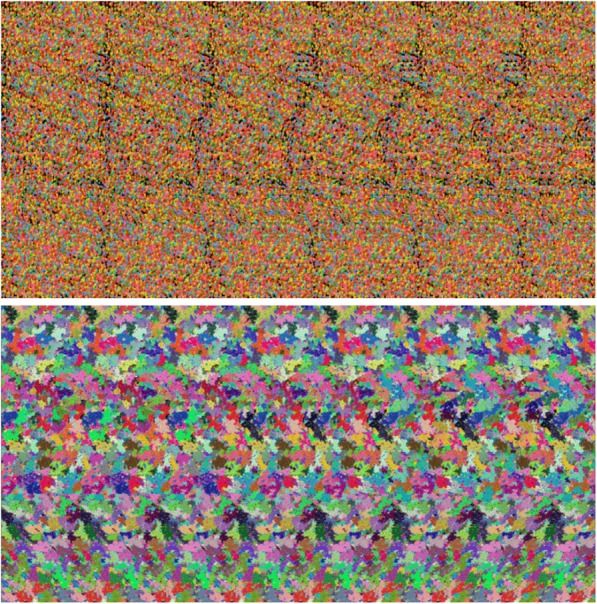
The two autostereograms used in the current study. The top autostereogram contains a shark [115], and the bottom a teapot [116]. The instructions for viewing are as follows: “Above is an autostereogram or Magic Eye© picture - to reveal the hidden 3D illusion, you must diverge your eyes (i.e. focus beyond the image). First, bring your face close to the page (so that you are almost touching it with your nose). The image should appear blurry. Focus as though you are looking through the image into the distance. Very slowly move away from the page until you begin to perceive depth in the image. At this point, hold very still and the hidden image will slowly appear”

The Autostereogram Self-Assessment (ASA) is a short four-item survey created for the present study where the participant self-assesses their autostereogram skill. Based upon short reports by Wilmer and Backus [59] and Cisarik, Davis, Kindy, and Butterfield [60], the questions asked the subject to identify two autostereograms (Fig. 1). The respondent was offered four possible choices plus an ‘I don’t know’ option. Correct answers were designated a score of 1, all other answers were given a 0. Respondents were also asked how difficult they found viewing the autostereograms (on a scale from 1 to 5, where 1 was extremely difficult and 5 very easy), and whether they had successfully perceived stereopsis in an autostereogram previously (‘yes’ answers were given a score of 1, all others a 0). Self-reported skill to perceive depth in autostereograms has been found to be predictive of stereoacuity, as measured by the TNO test (*r* = .45; [59, 60]).

### Motor skills

The Adult Developmental Coordination Disorder Checklist (ADC) is a validated screening tool for identifying the difficulties experienced by adults with DCD [57]. The ADC consists of three sub-scales; the first relates to difficulties that the individual experienced as a child (10 items). The second (10 items) and third sub-scales (20 items) relate to current difficulties. The second sub-scale focuses on the individual’s perception of their performance, whereas the third sub-scale relates to current feelings about their performance as reflected upon by others. All items are rated on a four-point scale (never, sometimes, frequently or always), resulting in possible scores ranging from 0 to 120. Recommended cut-off scores include 56 for “at risk of DCD” and 65 for “probable DCD” [57]. The latent structure of the ADC has not yet been confirmed using factor analysis.

### Social or autism-related traits

The Autism Spectrum Quotient (AQ) is a self-report questionnaire comprising of 50 statements [58]. It was designed as a measure of autistic characteristics in the general population. Although a 4-point response format is used, it is typically scored in a binary manner, where a response is scored as a one if it indicates an autistic trait and zero if this is not the case; this yields a score that can range from 0 to 50. Using this scoring approach, Baron-Cohen et al. [58] determined the optimal cut-off for identifying people with clinically significant levels of autistic traits to be 32 or above. The AQ can also be scored according to the 4-point response option [61, 62], which potentially yields a more sensitive index of ASD severity. In the current study, binary scoring was used to determine the proportion of participants that scored above the 32-point threshold mentioned previously. For all other analyses, including the exploratory factor analysis, the 4-point response was used. Past factor analyses of AQ items have been inconsistent, with studies finding two, three or four factors rather than five [63].

### Missing data

If a participant left more than 10% of responses across all items blank, the data were excluded from the analysis (*n* = 0; highest proportion of missing data for a single participant was 8.308%). The proportion of missing data for any individual questionnaire item ranged from 0 to 30%. Closer inspection of the pattern of missingness revealed that two items relating to driving ability in the Adult Developmental Coordination Disorder Checklist (“Did it take you longer than others to learn to drive?” and “If you are a driver, do you have difficulty parking a car?”) accounted for the highest amount of missing data (28.154% and 30% respectively). When the data from these questions were removed from the analysis, the highest proportion of missing data for a single item was reduced to 10.154%.

Although the number of subjects in the study was 650, 290 cases were missing a response for at least one item. Homoscedasticy of the data was tested using the TestMCARNormality function [64], which is part of the MissMech package in R. The test of homoscedasticy was rejected, indicating that the data was not missing completely at random (MCAR). The R package missForest [65] was used to impute the missing data. This has been demonstrated to introduce the least imputation error and has the smallest prediction difference from actual non-imputed values [66].

### Data analysis

Statistical analyses were performed using R 3.0.1. The relationship between scores on the ADC, AQ, ASA, and SSI were first examined using Pearson correlation analysis. The data were then randomly split into two equally sized groups (*n* = 325) to act as training and test data in a cross validation procedure. All items from all measures (minus the two ADC items mentioned above) within the training data set were subjected to exploratory factor analysis (EFA). Oblique rotation was specified for the EFA, given that the factors were expected to correlate with one another based on theoretical and empirical grounds [67]. Parallel analysis and Velicier’s Minimum Average Partial Test (available as part of the psych package) were used to determine the number of factors to retain. Factors were further interpreted if the grouping of the loading variables made conceptual sense. Given the fairly large sample size, items were considered to load onto a factor if their loading was ≥ .32 [68].

Cross-validation was then performed using the test data set, with the factors extracted using EFA being used to specify the factor structure for confirmatory factor analysis (CFA). In CFA, there is no single definitive indicator of model fit. The overall model fit was therefore assessed in terms of five measures from two perspectives: absolute fit and comparative fit to a base model, with index cut-offs (seen in brackets) informed by recommendations in the literature [69–72]. Absolute fit measures included the model chi-square/degrees of freedom (*χ2*/df; 3.0), standardised root mean square residual (SRMR; .08), and root mean square error of approximation (RMSEA; .06). The comparative measures were comparative fit index (CFI; .9) and Tucker-Lewis index (TLI; .9). Post hoc modification indices were applied to improve model fit. These indices were only used when modifications could be supported with theory as suggested by the literature; here, modifications consisted of allowing correlated residuals between items that loaded on to the same factor [73].

In the case where CFA fit indices indicated an adequate fit to the test data, bivariate correlations and subsequent moderation and mediation analyses in the form of structural equation modelling were conducted upon the extracted factor scores from the CFA to determine how they related to one another.

## Results

### Descriptive statistics

Tests of multi- and uni-variate normality indicated that the scores across all items did not meet the assumption of normality (Royston’s *H* test [74]; *H* = 11,580.473, *p* = < 0.001). For large sample sizes, significant results can be derived even in the case of a small deviation from normality [75].

All scales demonstrated acceptable internal consistency—see Table 1 for these and other descriptive data including the percentage of the total sample who met cut-off scores indicating clinically significant impairment for each measure. Of note is a higher incidence than would be expected of participants meeting cut-offs for clinically significant impairment for each standardised measure. These are higher incidences than would be expected from participants drawn from the general population (where DCD has a prevalence of approximately 5% [31]), ASD 1.1–2.4% [76, 77] and stereopsis deficit 40% [78]); however, one must be cautious when comparing rates of diagnosis in the clinic to questionnaire based estimates, see the “Discussion” section. It was not uncommon for participants who had a score above threshold for one measure to also score above threshold for at least one of the other measures (see Additional files 1, 2, and 3).

**Table 1:** Descriptive statistics for the Adult Developmental Coordination Disorder Checklist (ADC), Autism Spectrum Quotient (AQ), autostereogram self-assessment (ASA), and Stereopsis Screening Inventory (SSI) (*n* = 650). Clinically significant impairment is based on Coren and Hakstian [56], Kirby et al. [57], and Baron-Cohen e al. [58]

	M (SD)	Range	Cut-off scores indicating clinically significant impairment	% of participants meeting cut-off	Skewness	Kurtosis	Cronbach’s α
ADC	41.7 (21.97)	0–116	“DCD at risk” = 56–64	2.31%	0.75	3.09	0.94
			“Probable DCD” = ≥ 65	13.08%			
AQ	121.52 (22.5)	78–179	≥ 32	24.92%; note, 6 participants disclosed ASD diagnosis	0.29	2.16	0.91
ASA	3.6 (2.53)	1–8	Data not available	N/A	0.61	1.82	0.72
SSI	24.14 (9.28)	9–45	Moderate deficit = 17–29	34.62%	− 0.07	1.78	0.87
			Major deficit = ≥ 30	35.08%			

### Correlation of measure totals

Bivariate correlations of measure scores revealed a number of significant associations. A strong positive relationship was observed between AQ and ADC total scores (*r*(648) = 0.628, *p* = < 0.001), meaning that those with higher levels of autistic traits were also likely to exhibit higher levels of dyspraxic traits. Small-to-moderate positive correlations were observed between SSI, and both AQ (*r*(648) = 0.277, *p* = < 0.001) and ADC (*r*(648) = 0.268, *p* = < 0.001) scores, indicating that higher levels of autistic and dyspraxic traits were associated with an increased degree of stereoscopic deficit. A small negative relationship was also observed between ASA and ADC scores (*r*(648) = − 0.106, *p* = 0.007), denoting that those with increased dyspraxic traits tended to be worse at perceiving autostereograms. No significant relationship was found between ASA and either AQ (*p* = 0.056) or (surprisingly) SSI scores (*p* = 0.502).

### Exploratory factor analysis

The aim of this study was to assess the existence of latent variables, thus EFA was used to determine the dimensional structure of pooled items across the four measures previously described.

For the training dataset (*n* = 325) the Kaiser-Meyer-Olkin coefficient of sampling adequacy was good (.857; .6 is recommended by Cerny and Kaiser [79]) and Bartlett’s test of sphericity [80] was significant (*χ*^*2*^ (5151) = 17,439.845, *p* = < 0.001), indicating that the data were suitable for factor analysis. Parallel analysis [81] and Velicer’s minimum average partial test [82] recommended that 10 factors be extracted from the data [83, 84]. Factor loadings were calculated using principal axis factoring with oblmin (oblique) rotation on 102 of 104 Likert scale questions across all four measures (omitting the two items of the ADC which had a high proportion of missing data, see the “Methods” section) and are shown in Table 2. Labels have been provided for the 10 extracted factors, based on an interpretation of the items that constitute them; ‘social skill’, ‘stereopsis’, ‘attention to detail’, ‘fine motor skill’, ‘organisation’, ‘Magic Eye proficiency’, ‘isolation due to motor proficiency’, ‘coordination’, ‘imagination’, and ‘multitasking’. All items loading on to these factors are shown in Table 2.

**Table 2:** Factor loadings of a 10-factor EFA solution for items pooled across all measures. Principal axis factoring, oblmin rotation. Loadings below .32 (which explain less than 10% of the variance in that item) are not highlighted and are considered to be negligible loadings for the purposes of analysis

Measure	Item	Social	Stereo	Detail	Fine motor	Org	Magic Eye	Isolation	Coord	Imagine	Multi
AQ	Enjoy social chitchat	**0.70**	0.01	−0.06	−0.09	−0.06	−0.03	−0.04	0.07	0.01	0.08
AQ	Good at social chitchat	**0.70**	0.04	0.02	−0.05	0.03	0.12	0.03	0.01	−0.05	−0.03
AQ	Find social situations easy	**0.67**	−0.05	0.07	−0.01	0.01	0.03	0.01	0.00	0.02	−0.08
AQ	Prefer people over things	**0.59**	−0.01	−0.12	−0.04	0.02	0.04	−0.01	−0.04	0.04	0.09
AQ	Enjoy social occasions	**0.56**	−0.07	−0.10	−0.14	0.02	−0.02	−0.10	−0.09	0.08	0.01
AQ	Enjoy meeting new people	**0.53**	−0.07	−0.12	0.02	0.00	0.02	−0.04	−0.20	0.05	0.04
ADC	Choose to spend leisure time on own	**−0.48**	0.05	0.09	0.20	−0.02	0.02	0.25	−0.01	0.01	0.06
AQ	Easily keep track of several conversations	**0.44**	−0.03	0.15	0.00	−0.08	−0.01	0.01	0.07	0.19	−0.19
AQ	Can work out what someone is feeling from their face	**0.43**	−0.02	0.00	−0.06	−0.08	−0.01	−0.02	0.06	**0.34**	−0.10
AQ	Prefer to do things with others	**0.43**	0.05	0.02	0.13	−0.11	−0.02	−0.11	0.02	−0.22	−0.05
AQ	Find it hard to make new friends	**−0.41**	−0.06	0.30	−0.05	−0.04	−0.06	0.20	0.15	−0.16	−0.06
AQ	New situations bring on anxiety	**−0.35**	0.08	0.11	−0.11	0.01	0.06	0.17	0.09	−0.08	0.24
AQ	Don’t know how to keep conversation going	**−0.33**	−0.04	0.22	0.04	0.02	−0.09	0.11	−0.00	−0.25	0.11
AQ	Can easily ‘read between the lines’	**0.32**	−0.03	0.02	−0.10	−0.03	0.10	0.06	0.03	0.31	−0.14
SSI	Do you think you need glasses	0.03	**0.94**	0.02	0.05	0.01	−0.01	−0.03	−0.03	0.03	0.01
SSI	Glasses/contact lens wearer	−0.05	**0.90**	−0.00	−0.00	0.01	0.02	−0.02	−0.02	0.02	−0.00
SSI	W/out correction, clearness of vision in LEFT eye	0.08	**0.89**	0.02	0.03	−0.04	−0.01	0.07	−0.05	−0.04	0.05
SSI	W/out correction, clearness of vision in RIGHT eye	−0.08	**0.88**	−0.06	−0.03	0.00	0.02	−0.02	0.03	0.06	−0.03
SSI	Vision as good as other people’s	0.06	**0.87**	0.02	−0.03	0.03	−0.04	−0.00	−0.00	−0.06	−0.01
SSI	Correction needed for reading	−0.02	**0.53**	0.01	−0.04	−0.02	0.05	−0.09	0.21	−0.05	−0.08
AQ	Notice patterns in things all the time	−0.13	0.05	**0.67**	−0.08	0.10	0.02	−0.03	−0.08	0.05	−0.05
AQ	Notice car number plates or similar	−0.01	0.02	**0.56**	−0.03	0.04	0.06	0.03	−0.01	−0.02	−0.07
AQ	Tend to notice details that others do not	−0.14	−0.02	**0.55**	0.12	0.06	−0.00	−0.10	0.01	0.30	−0.09
AQ	Strong interests, get upset if can’t pursue	0.01	0.04	**0.55**	0.14	−0.02	−0.04	0.05	−0.03	0.03	0.16
AQ	Notice small sounds	−0.13	0.07	**0.47**	0.07	0.07	0.06	0.01	0.05	0.18	0.07
AQ	Get strongly absorbed in one thing	−0.24	0.12	**0.46**	−0.06	0.18	−0.02	−0.02	−0.01	−0.03	−0.02
AQ	Enjoy collecting information about categories	−0.01	−0.07	**0.45**	0.13	−0.08	0.02	0.04	0.09	−0.07	0.05
AQ	Repetitive topic of conversation	0.06	0.03	**0.45**	0.11	0.03	−0.01	0.13	−0.01	−0.18	0.01
AQ	Tend to dominate conversation	0.18	0.03	**0.43**	0.05	−0.06	0.06	0.09	−0.02	−0.04	0.12
AQ	Fascinated by numbers	−0.07	−0.05	**0.41**	0.07	0.03	0.03	−0.07	0.04	−0.08	−0.06
AQ	Difficult to work out people’s intentions	−0.06	0.06	**0.39**	−0.00	0.04	−0.03	0.06	−0.00	−0.29	0.16
AQ	Difficulty imagining being someone else	0.08	−0.09	**0.37**	0.14	−0.01	−0.12	0.09	−0.06	−0.16	0.19
AQ	Say impolite things without realising	0.09	−0.02	**0.36**	0.14	−0.12	−0.03	0.12	0.02	−0.04	0.25
AQ	Difficulty speaking in turns on phone	−0.01	0.03	**0.35**	0.13	0.07	−0.05	0.10	0.09	−0.05	0.10
AQ	Difficultly working out characters’ intentions in story	0.12	0.01	**0.34**	0.06	−0.04	0.14	0.13	0.13	−0.24	0.02
ADC	Others find it difficult to read your writing	0.01	0.04	0.00	**0.79**	−0.07	0.01	0.05	−0.07	0.01	−0.06
ADC	Difficulty with writing neatly AND quickly	−0.06	0.02	−0.03	**0.73**	0.03	−0.04	0.02	−0.02	0.03	0.09
ADC	Difficulty with neat writing when child	−0.07	−0.01	0.07	**0.70**	0.13	−0.04	0.07	−0.11	0.00	0.00
ADC	Difficulties reading own writing	−0.04	0.07	−0.05	**0.66**	−0.04	0.03	−0.08	0.18	−0.01	−0.15
ADC	Difficulties with writing as fast as peers	0.00	−0.04	0.07	**0.65**	0.06	0.03	−0.04	0.09	−0.12	0.05
ADC	Difficulty with fast writing as child	0.04	−0.06	0.06	**0.62**	0.15	0.03	0.01	0.06	−0.07	0.06
ADC	Difficulty copying without mistakes	−0.09	−0.06	−0.09	**0.43**	0.10	−0.02	−0.15	0.27	0.04	0.08
ADC	Difficulty with organisation	−0.04	0.07	−0.06	0.14	**0.71**	0.07	−0.09	0.02	−0.00	−0.03
ADC	Difficulties with organisation as child	−0.01	0.09	−0.05	0.06	**0.68**	−0.02	0.10	−0.06	−0.01	−0.09
ADC	Others call you disorganised	0.06	0.00	0.11	0.08	**0.66**	0.09	0.01	0.02	−0.04	0.00
ADC	Tend to lose possessions	0.01	−0.09	−0.03	0.04	**0.58**	−0.04	0.15	0.03	0.05	0.03
ADC	Difficulty sitting still	−0.08	−0.07	0.23	0.05	**0.52**	−0.04	−0.02	−0.05	0.07	0.18
ADC	Difficulty planning ahead	−0.08	0.03	0.09	−0.02	**0.48**	0.04	−0.14	0.02	−0.18	0.29
ADC	Bump into, spill, or break things	−0.00	−0.07	−0.02	−0.04	**0.43**	−0.10	**0.41**	0.19	0.04	0.02
ADC	Difficulty managing money	−0.02	−0.04	0.05	0.01	**0.43**	0.04	−0.17	0.23	−0.03	0.15
ADC	Can lose attention in certain situations	−0.02	0.05	0.12	0.02	**0.42**	−0.02	−0.05	0.04	−0.07	0.31
ADC	Bumped into objects more than other children	0.03	−0.07	0.09	0.05	**0.38**	−0.13	**0.36**	0.20	−0.02	−0.03
MEA	Identify shape in autostereogram [shark]	−0.05	0.00	−0.03	0.01	−0.00	**0.91**	0.04	0.01	0.01	0.02
MEA	Identify shape in autostereogram [teapot]	0.05	−0.00	0.01	−0.03	0.04	**0.91**	0.00	0.06	−0.06	−0.03
MEA	Ease of perceiving shapes in autostereograms above	0.03	−0.02	0.03	0.04	0.01	**0.85**	0.01	−0.03	0.06	0.04
MEA	Previous successful completion of autostereogram	−0.09	0.10	0.09	−0.01	−0.01	**0.35**	0.22	−0.15	0.04	−0.04
ADC	If do sport, likely to be on your own	−0.16	−0.01	0.04	0.05	−0.04	0.06	**0.63**	−0.08	−0.04	−0.03
ADC	Avoid team games/sports	−0.18	0.11	0.05	0.02	0.00	0.09	**0.60**	0.03	0.02	0.08
ADC	Difficulties playing team games as child	−0.04	0.13	−0.01	0.14	0.02	0.01	**0.47**	0.17	−0.05	0.12
ADC	Others commented on clumsiness as child	0.13	0.02	0.06	0.01	**0.35**	−0.16	**0.44**	0.23	−0.04	−0.06
ADC	Difficulties with hobbies requiring good coordination	−0.03	0.10	−0.10	0.02	0.09	−0.03	0.25	**0.57**	0.03	0.05
ADC	Difficulties eating with utensils	−0.03	−0.08	0.06	0.08	0.01	0.05	−0.09	**0.57**	−0.09	0.06
ADC	Self-care difficulties	−0.08	0.02	0.05	0.19	0.07	−0.08	−0.01	**0.48**	−0.08	0.08
ADC	Avoid hobbies that require good coordination	−0.02	0.13	−0.08	0.02	−0.02	−0.02	**0.36**	**0.45**	−0.03	0.15
AQ	Can easily imagine what characters in story look like	0.00	−0.01	0.08	−0.16	−0.08	0.08	−0.00	0.05	**0.52**	−0.00
AQ	Easily play games with children involving pretending	0.28	−0.07	−0.05	−0.10	0.05	0.05	0.08	−0.15	**0.43**	0.14
AQ	Easy to create a picture using imagination	0.01	−0.01	0.25	−0.03	−0.08	0.08	−0.09	−0.01	**0.42**	0.02
AQ	Is a good diplomat	0.28	0.02	−0.00	0.05	−0.08	−0.00	−0.11	−0.01	**0.37**	−0.05
AQ	Making up stories is easy	0.06	0.00	0.23	−0.08	0.08	0.05	0.12	−0.08	**0.37**	−0.02
ADC	Difficulty performing concurrent tasks	−0.11	0.04	0.02	0.20	0.10	−0.02	−0.05	0.24	0.08	**0.41**
ADC	Difficulty with distance estimation	0.06	0.12	0.02	−0.06	0.14	−0.07	0.23	0.13	−0.04	**0.39**
AQ	Easy to do more than one thing at once	0.28	0.04	0.04	−0.05	−0.02	0.02	0.03	−0.09	0.13	**−0.34**
ADC	Difficulty with navigation	0.00	0.09	−0.09	0.09	0.03	−0.07	0.18	0.16	−0.06	0.32
ADC	Difficulty packing suitcase to go away	0.03	−0.05	0.13	0.09	0.21	0.00	−0.05	0.26	−0.02	0.29
ADC	Difficulty learning to ride bike as child	0.06	0.05	0.04	0.12	0.04	−0.03	0.25	0.13	0.02	0.28
ADC	Difficulty preparing meal from scratch	−0.03	−0.02	0.14	−0.01	0.04	−0.05	−0.09	0.27	−0.16	0.25
AQ	Prefer to do things the same way over and over	−0.05	0.00	0.30	0.16	−0.10	−0.06	0.11	0.02	0.04	0.25
AQ	Know if someone listening to me is getting bored	0.27	−0.05	−0.10	0.03	−0.05	−0.04	−0.07	−0.07	0.29	−0.23
ADC	Difficulties with self-care when child	0.10	−0.03	0.01	0.18	0.15	−0.12	0.18	0.26	0.07	0.22
ADC	Difficulty folding and putting away clothes	0.11	0.07	0.13	0.28	0.24	0.00	−0.01	0.20	−0.03	0.22
AQ	Not upset if daily routine is disturbed	0.27	−0.12	−0.06	0.05	0.06	0.04	−0.05	−0.04	0.04	−0.21
AQ	Enjoy doing things spontaneously	0.29	−0.16	−0.09	0.08	0.15	0.10	−0.13	−0.18	0.06	−0.21
AQ	Quickly go back to previous activity after interruption	0.19	−0.04	−0.01	0.04	−0.10	0.09	0.08	−0.01	0.23	−0.21
ADC	Slower at getting ready	−0.00	0.07	0.08	0.17	0.28	0.07	−0.03	0.12	−0.13	0.20
AQ	When younger, enjoyed pretend games with others	0.10	0.04	−0.25	−0.08	0.08	0.10	0.03	−0.20	0.29	0.19
AQ	Carefully plan any activities participated in	−0.13	0.06	0.31	0.07	−0.27	−0.07	0.09	0.14	0.16	0.19
SSI	Book too close to eyes when reading	−0.09	0.29	0.03	−0.09	0.09	−0.05	0.18	0.09	−0.09	−0.18
AQ	Not very good at remembering phone numbers	−0.01	0.04	−0.24	0.19	−0.08	−0.03	0.14	0.01	0.11	0.18
SSI	Experience temporary loss of vision	−0.05	0.16	0.03	0.04	0.13	−0.15	0.00	0.30	0.10	−0.17
AQ	Not good at remembering people’s date of birth	−0.09	−0.02	−0.15	0.20	0.11	0.04	0.12	−0.17	0.09	0.17
AQ	Don’t enjoy reading fiction	0.10	−0.02	0.07	0.03	0.02	0.04	0.06	−0.01	−0.06	0.15
AQ	Rather go to library than a party	−0.24	0.12	0.22	0.08	−0.05	0.04	0.25	0.09	0.08	−0.13
AQ	Concentrate on whole rather than parts	0.13	−0.04	−0.23	−0.05	0.09	0.02	0.00	0.07	0.11	−0.11
ADC	Do you avoid going to clubs/dancing	−0.30	0.09	0.15	0.08	0.06	0.01	0.25	−0.06	−0.04	−0.06
AQ	Last to understand the point of a joke	0.14	0.00	0.32	0.01	0.05	−0.14	0.09	0.16	−0.12	0.05
AQ	Fascinated by dates	0.07	0.03	0.26	−0.02	−0.06	0.10	−0.03	0.14	−0.03	−0.05
AQ	Don’t notice small changes	0.01	0.12	0.02	0.20	−0.02	−0.00	0.11	−0.13	−0.20	0.03
AQ	Rather go to the theater than to a museum	0.29	0.01	−0.09	−0.10	−0.05	−0.05	−0.18	0.18	0.04	0.03
ADC	Difficulties playing music instrument when child	0.04	−0.03	−0.03	0.21	0.18	−0.17	0.18	0.15	−0.01	0.01
SSI	Difference between items 8 and 9	0.01	0.05	0.08	−0.01	−0.04	−0.10	0.04	0.03	0.02	−0.01
SSI	Eyes feel ‘tired’	0.09	0.30	−0.02	0.10	0.06	−0.07	−0.03	0.25	0.04	−0.00

### Confirmatory factor analysis

The factor structure suggested by EFA was cross-validated by means of CFA, using the lavaan package. The ‘test’ data (*n* = 325) were analysed using the MLR estimator, which is robust to the non-normality of the observed variables [85]. In the first model, items (indicators in CFA terminology) which had a sufficiently high factor loading in the initial EFA (≥ .32) were estimated as free parameters; all other items were fixed to zero. The factors (or latent variables) were allowed to covary freely. Though the initial model showed a reasonable fit on some of the indicators, it did not meet criteria for acceptable fit for the comparative fit indices (*χ*^*2*^/df = 2.013, CFI = 0.782, TLI = 0.771, SRMR = 0.08, RMSEA = 0.056). This is to be expected, as the initial model to be tested through CFA had more stringent restrictions than the factor model obtained through EFA, where no factor loadings were fixed to zero. In studies using cross-validation procedures such as those performed here, it is recommended that a less constrained model is tested where some parameters are freed [86]. Modification indices were allowed in the creation of an adjusted model, though with restrictions upon which changes could be reasonably made to the initial model.

After modification indices were applied, where the residuals between indicators loading on to the same latent variable were allowed to correlate with one another if this significantly improved the fit of the model, all indices indicated an acceptable fit (*χ*^*2*^/df = 1.485, CFI = 0.899, TLI = 0.89, SRMR = 0.069, RMSEA = 0.039). A scaled chi-square difference test [87] showed that this modification-index-adjusted model exhibited a significantly better fit compared to the initial model (∆*χ*^*2*^(88) = 1261.05, *p* = < 0.001). Factor scores were calculated from the adjusted CFA model using simple regression [88] for each participant. These scores were then used to perform mediation analyses in order to better understand the relationships between the factors or latent variables.

### Mediation

Factors were only included in this aspect of the analysis where strong a-priori hypotheses could be made: stereopsis, Magic Eye proficiency, fine motor skill, coordination, isolation due to motor proficiency, and social skill factor scores were retained. As can be seen in Table 3, the majority of these factors showed medium-to-large correlations with one another. All mediation analyses reported here were performed using lavaan’s structural equation modelling (SEM) framework.

**Table 3:** Spearman correlation coefficients for factor scores extracted using confirmatory factor analysis (CFA)

	Stereo	Magic Eye	Social	Isolation	Coord
Magic Eye	0.14*	–			
Social	− 0.34***	0.05	–		
Isolation	0.21**	− 0.12	− 0.53***	–	
Coord	0.20**	− 0.18**	− 0.55***	0.86***	–
Fine motor	0.19**	− 0.08	− 0.54***	0.64***	0.67***

Significant relationships are indicated by asterisks. *p < 0.05, **p < 0.01, ***p < .001. Significance values Bonferroni corrected in order to adjust for multiple comparisons

### Motor skills may mediate the link between stereopsis and social skills

Fine motor skill, coordination, and isolation due to motor proficiency were entered into a multiple mediation analysis to investigate the relationship between stereopsis impairment and reduced social ability. A significant total effect of stereopsis on social skills emerged, β = − 0.312, *z* = − 6.143, *p* = < 0.001. When dividing this total effect into the direct effect of stereopsis, and the total indirect effects of all three mediators, the direct effect of stereopsis remained significant after adjusting for all three mediators, β = − 0.216, *z* = − 4.615, *p* = < 0.001. The total indirect effect was also significant, β = − 0.096, *z* = − 3.441, *p* = < 0.001. Of the three mediator variables, only fine motor skill contributed significantly to the indirect effect of stereopsis upon social skills (12.436% of the total effect; β = − 0.039, *z* = − 2.276, *p* = 0.02). Neither coordination nor isolation exhibited a significant amount of mediation (*p* = 0.061 and 0.178, respectively).

### Motor skills mediate the link between stereopsis and isolation

To investigate why individuals with worse stereopsis reported increased isolation due to motor proficiency, a multiple mediation analysis was performed with the mediator variables being fine motor skill and coordination. A significant total effect of stereopsis on isolation emerged, β = 0.179, *z* = 3.47, *p* = < 0.001. When dividing this total effect into the direct effect of stereopsis, and the total indirect effects of both mediators, the direct effect of stereopsis was no longer significant, β = 0.02, *z* = 0.772, *p* = 0.44, but the total indirect effect was significant, β = 0.158, *z* = 3.545, *p* = < 0.001. Both mediator variables contributed significantly to the indirect effect of stereopsis upon isolation due to motor proficiency, though coordination exhibited a greater proportion of mediation (75.3% of the total effect; β = 0.134, *z* = 3.471, *p* = < 0.001) than fine motor skills (13.273% of the total effect; β = 0.024, *z* = 2.294, *p* = 0.02).

### Isolation may mediate the link between coordination/fine motor skills and social skills

Two final mediation models indicated that isolation due to motor proficiency was a significant mediator both in the relationship between coordination and social skills (39.772% of the total effect; β = − 0.211, *z* = − 2.533, *p* = 0.01) and fine motor skill and social skills (40.941% of the total effect; β = − 0.214, *z* = − 4.477, *p* = < 0.001). Partial mediation occurred in both cases, as coordination and fine motor skill were still significant predictors of social skills after adjusting for the indirect effect of isolation (coordination: β = − 0.32, *z* = − 3.647, *p* = < 0.001, fine motor skills: β = − 0.309, *z* = − 4.605, *p* = < 0.001).

### Path analysis

**Fig. 2:**
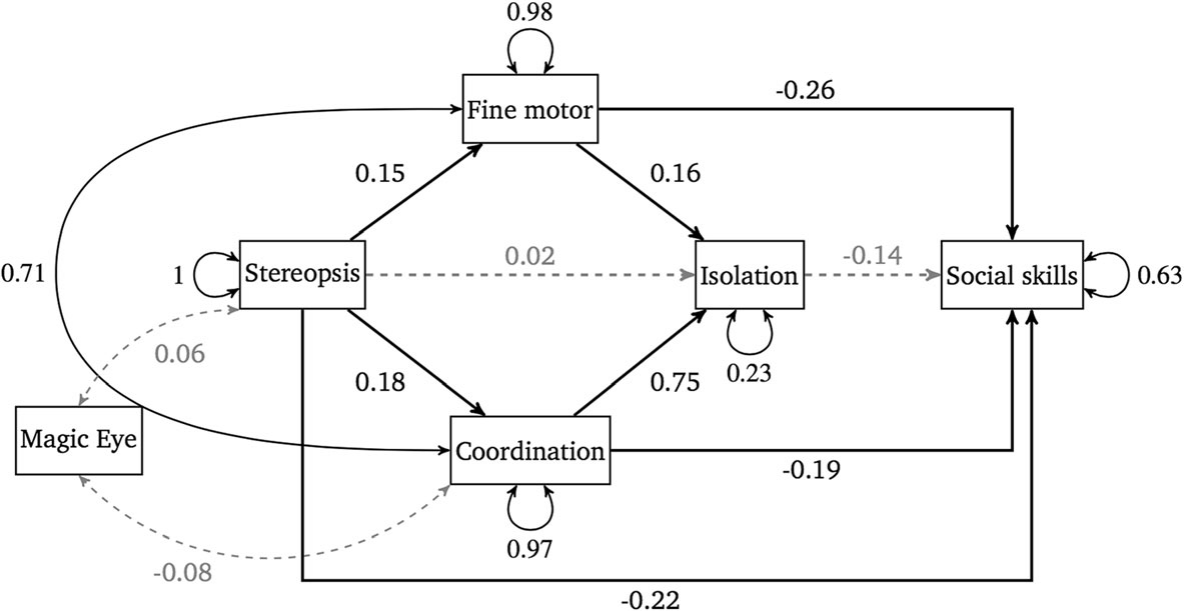
Path model with standardised estimates, created as an amalgamation of the mediation analyses. Paths with solid arrows signify a significant predictive relationship, whereas dashed arrows indicate a non-significant relationship

The above mediation models were aggregated into a larger path model. This final model included relationships with Magic Eye proficiency as detailed in Table 3. This model had a good fit, with *χ*^*2*^/df = 0.418, CFI = 1, TLI = 1.011, SRMR = 0.017, and RMSEA = < 0.001. The results of the path analysis with standardised regression coefficients are presented in Fig. 2. The relationship between stereopsis and social skills, as well as stereopsis and isolation (both mediated by fine motor skill and coordination) held in this larger model. The effect of isolation due to motor proficiency acting as a mediator between fine motor skill/coordination and social skills did not hold in this larger model. Whilst fine motor skill and coordination were responsible for full mediation of the relationship between stereopsis and isolation due to motor proficiency, there was no serial mediation from the fine motor/coordination variables to social skills via the isolation variable. Finally, Magic Eye proficiency was not a significant independent variable within the context of the path model.

## Discussion

The aim of the present study was to explore the relationship between stereopsis, motor ability, and social skills in a sample of adults. The current research builds upon prior work by investigating whether the impact of motor impairment upon social functioning persists in adulthood, as well as incorporating a variable, stereopsis, which may underlie deficits in motor ability and thus have an impact upon social skill. The results indicated that impaired stereopsis both directly and indirectly affected social skills, in the latter case through mediation by coordination and fine motor skill. Additionally, both fine motor skill and coordination fully mediated the relationship between stereopsis and isolation due to motor proficiency, with coordination explaining much larger proportion of variance. However, in the full model, isolation due to motor proficiency did not have a significant relationship with social skills.

Overall, the results of this study suggest that stereopsis impairment can affect both motor skill proficiency and social skills. Additionally, as the final aggregate path model was a good fit for the data, preliminary support is provided for the validity of the causal pathways in the model.

### Associations between stereopsis, motor skills, and isolation

The findings reported here support the hypothesis of links between impaired stereopsis and both fine and gross motor skills. In the current study, there was also a relationship between stereopsis impairment and coordination/daily living skills. Little previous research has looked at this more functional consequence of impaired stereopsis. It has been observed that the sensation of depth afforded by binocular viewing is important for certain gross motor skills, such as obstacle avoidance whilst walking [18] and intercepting thrown objects [89], but only two studies have specifically looked at the contribution of reduced stereopsis to daily living skills.

In a group of older individuals (aged 65 years), Kuang, Hsu, Chou, Tsai, and Chou [23] found no effect of stereopsis on daily living tasks such as cooking and writing, but they did observe that those with poor stereopsis exhibited a reduction in reported energy/vitality, suggesting that more effort may be required to accomplish daily living tasks. Cao and Markowitz [90] noted that in a group of older subjects (aged 50 years) with age-related macular degeneration, those with reduced stereopsis experienced difficulty with visual motor skills required for daily living. The observers in the current study were younger than the groups surveyed by Kuang et al. [23] and Cao and Markowitz [90], with 92.6% of the participants who disclosed their age being under 60 years old, thus, here we extend the finding of a relationship between stereopsis and daily living skills to younger and middle-aged adult populations.

Whilst there was a relationship between stereopsis and both types of motor proficiency, the size of this effect was small within the context of the path model. A much stronger association was present between fine motor skill/coordination and isolation. Of these two facets of motor skill that showed links with isolation, it was coordination/daily living skills (which require gross motor ability) that exhibited the largest amount of mediation between stereopsis and isolation. Whilst there is already evidence that motor ability correlates with feelings of isolation and social standing with peers [8–10, 40, 91], these studies do not tend to differentiate between fine and gross motor skill. Future work might look at whether social isolation is due to simple impairment in gross motor skills or if it might be more specifically attributed to a reduction in daily living skills; such knowledge would allow more targeted treatment (such as physical therapy for gross motor skills versus occupational therapy for daily living skills).

### The impact of impaired stereopsis on social skills

Impaired stereopsis may affect social skill by causing a reduction in general motor ability. The current results are consistent with those who have previously found an association between motor proficiency and social competence [8, 34–37, 92, 93]. Whilst fine motor skill and coordination did mediate the relationship between stereopsis and social skill, this effect was only partial (the mediation model accounted for around a third of the variance in the relationship between stereopsis and social skill). Fine motor skill, coordination, and stereopsis all exhibited a similar strength of effect in their relationship with social skill. That the mediators between stereopsis and social skill accounted for only a small amount of variance suggests that there are other unmeasured factors that play a part in the relationship between impaired stereopsis and reduced social skill. The findings here suggest that stereopsis may prove useful in other, as yet unexplored, domains related to social interaction—for instance, the estimation of interpersonal distance.

It is interesting to speculate on the underlying mechanisms between impaired stereopsis and social skills. Theories from autism research have attempted to link visual and social abilities, via a common, generalised, cause [94]. Pellicano and Burr [95] use a Bayesian framework to argue that flattened priors may account for the changes in autism. They argue that many of the traits underlying autism are related to a failure to update perception from prior experience. It is not clear whether this general deficit extends to depth and stereo-disparity processing, however, since people with and without autism integrate depth cues similarly [51]. An alternative theory proposes that autistic individuals have enhanced perceptual function (EPF) [96] in early associative areas of sensory processing (e.g. visual discrimination), resulting in greater locally oriented processing. This account suggests that higher-order processing is not always engaged or mandatory in autism, when a task can be carried out using lower-level perceptual processing. Therefore, when presented with complex and fast moving social stimuli (e.g. a person speaking), a strong focus on low-level perceptual features may result in information overload and an inability to attend to the relevant visual cues. This account appears to conflict with the current results in that we find a link between impaired, rather than enhanced, perceptual function and social isolation. It is worth noting, however, that the perceptual losses described here are likely to predominantly come from issues at the earliest stages of perceptual processing such as lack of eye alignment (strabismus or squint) as well as, possibly, more neurological deficits. The links proposed between EPF and social abilities are usually described as more complex cognitive biases which are not necessarily linked to depth perception [51]. It is important, therefore, to consider the impact of both peripheral perceptual and cognitive differences to understand social behaviour.

An alternative explanation for the link between stereopsis and social abilities and behaviours is that the link is environmentally mediated and is due to selective reinforcement of behaviours. As discussed in the introduction, stereopsis cues to depth are most useful in peri-personal space [20] and thus would be useful for judging social distance and interpersonal space. Furthermore, optical conditions that impair depth perception, such as amblyopia or strabismus, have also been linked to social exclusion and reduced quality of life measures [21, 97]. Under this explanation, poor stereopsis reduces the opportunity to develop social skills, especially in childhood, and this extends into adulthood. This explanation must also be viewed with caution, not least because it is not clear whether the deficits in those with strabismus are due to the condition itself or the treatment [98]. Further work to test the whether the predictions of clinical models extend to the general population is necessary.

### Isolation due to motor proficiency does not predict general social ability

In contrast to previous research which has established that perceived and/or actual social isolation causes individuals to change their behaviour and have lower-quality social interactions [99–101], we did not find that isolation due to motor proficiency significantly predicted social skill in the full path model. It is likely that motor ability (represented by the fine motor skill and coordination variables) is responsible for this relationship, especially considering the items that constitute the isolation factor all relate to motor proficiency, specifically in the context of sport and team games. When isolation is characterised more fully, including indicators such as social network size, participation in a range of social activities (not just those that require motor proficiency), and perceived lack of social support, the relationship between isolation and social ability is likely to hold true.

### Limitations

It is assumed that the greater correlations between the AQ and ADC scores compared to the SSI score, and the motor (fine motor and coordination) and social skills factor scores compared to the stereopsis factor score reflect a greater interdependence of social and motor skills in development. However, it is possible that the stronger correlation may be an artefact of the questionnaires used, with the two questionnaires with the largest number of questions and covering a range of domains (the AQ and ADC) correlating most strongly. Coren and Hakstian [56] have established that whilst the SSI has a relatively high specificity, the sensitivity is relatively poor (59.7%). A lab- or clinic-derived measure of stereoacuity might highlight relatively larger (or smaller, dependent on whether the stereopsis factor extracted in the current study actually measures this function) correlations with social and motor skills. Related to this point, whilst self-report questionnaires are easy to administer to a large number of individuals, their subjective nature may result in biased responses [102]. However, the questionnaires used in this study are well standardised and have demonstrable construct validity. The ADC and AQ in particular are commonly employed as research and screening tools.

It is interesting to note that our correlations between stereoacuity and Magic Eye proficiency factor scores were relatively low, although significant (see Table 3); there was no significant correlation between the ASA and SSI total scores. For the factor scores, our correlation value is slightly lower than the value of 0.34 previously found by Wilmer and Backus [59] in a similar comparison. Our comparison was slightly different to that previous study in that we asked people to report their difficulty resolving the autostereogram image, which could account for some of the difference. Furthermore, to be successful with the autostereogram, participants require good near convergence which is not covered by the SSI stereoacuity measure [103]. As above, conclusions regarding stereoacuity based on questionnaires must be cautious until they are followed up with controlled clinic or laboratory measurement.

Our sample was non-stratified and was biased towards university students; however, we note that the number of participants sampled was markedly higher than the majority of studies, which administer the AQ in a nonclinical sample (which is by far the most commonly reported questionnaire of the ones used in the current study [104]). The recruitment strategies used for the current study are similar to the trends noted for other research involving the AQ, including part of the participant sample being drawn from participant databases maintained by universities, and the use of online survey tools to reach a broader audience [104].

There was a relatively high proportion of participants who surpassed the threshold for clinically significant levels of impairment across all of the standardised questionnaires we used. This may be due to self-selection bias as the study was advertised as a “survey on correlations between visual ability, coordination, and autistic traits”. Individuals who perceived themselves as clumsy, having poor social skills, or problems with visual perception may have been more likely to take part, creating an opportunistic selection bias. The particularly high proportion of participants meeting or exceeding the AQ cut-off may reflect the large proportion of individuals either pursuing a STEM degree or in a STEM career, who are more likely to score higher on the AQ than those in non-STEM education or career paths [105]. Furthermore, whilst only six participants disclosed a diagnosis of autism or Asperger’s syndrome, more specified that they were first-degree relatives of someone with the condition. It is thought that autistic traits may be expressed to a greater degree in close relatives of people with an ASD, even though they might not meet the criteria for clinical diagnosis [106], a concept termed the broader autism phenotype [107–109]. However, our final sample showed a broad range of individual differences in the scores of the SSI, AQ, and ADC, indicating that whilst self-selection bias may have occurred, there was still sufficient variability in the data to allow us to conduct our analyses.

### Clinical implications

The first and most important clinical implication of this study is that visual deficits such as reduced stereopsis can have far reaching implications on behaviour. Interventions to improve stereopsis itself have had limited success but are probably not sufficiently developed to be recommended to ameliorate the issues described here [110–112]. Nevertheless, it would be important to address the sensory and motor issues in the clinic. Stereopsis is not the only cue to depth; many other cues to depth such as texture gradient, size, occlusion are available. Those with reduced stereopsis are likely to use cues differently to those with good or normal stereopsis [51]. For many tasks, simply adding a pattern to a surface can improve the ability to judge and use depth cues, by providing more size and texture gradient cues. This can improve activities such as walking and stepping [113, 114] so may have implications for problems of dexterity and clumsiness. For social skills, it is possible that therapies which guide those with reduced stereopsis to use alternative cues to judge critical distances such as interpersonal distance might be particularly effective. For instance, training people to use rules such as keeping an arm’s length away rather than relying on implicit cues might be helpful. Finally, it is possible that the link between stereopsis and social skill is because the perceptual deficits reduce the likelihood that people engage in social activities. Thus, in this case, it would be the clinician’s role to support the child (or adult) to find social activities which are not affected by a loss of stereopsis.

## Conclusions

This study has demonstrated the presence of a relationship between stereopsis, motor ability, and social skill. Using a large group of adults, this work complements research previously conducted with children, in addition to providing evidence for an underlying contributor to impairment in both motor and social skill. Preliminary support for causal pathways between stereopsis, motor ability, and social skill has been provided, but further evidence is needed to clarify the mechanisms responsible, especially in clinical populations. The repercussions of poor stereopsis have been demonstrated to be far-reaching, limiting not only motor skill, but also social competence.

## Additional files

Additional file 1: **Table S1.** A detailed breakdown of self-reported occupation, including faculty for those in education (where available) and sector for those in employment. (DOCX 25 kb)

Additional file 2: **Table S2.** The psychiatric or organic illnesses self-disclosed in the feedback section of the questionnaire. Note that diagnoses were not collected routinely as part of the demographical data. The data below represent a number of co-morbidities: 32 diagnoses were disclosed by 24 participants (3.7% of the sample). (DOCX 24 kb)

Additional file 3: **Figure S1.** Depicted is a 3-set Venn diagram where the size of the ovals indicates relative magnitude and the numbers within portray the number of participants who scored above threshold on th(at|ose) measure(s). Note that for the SSI, the higher threshold boundary indicating major stereopsis deficit was used. Descriptive statistics for questionnaire responses: It was not uncommon for participants who had a score above threshold for one measure to also score above threshold for at least one of the other measures. The most substantial amount of overlap between measures was for the AQ and the SSI, with 10.615% of the total participant sample scoring above threshold on both of these measures (note that the higher SSI threshold indicating major stereopsis deficit was used in this case). However, the largest degree of overlap was between the ADC and AQ, with 71.765% of participants who met the threshold for ‘probable developmental coordination disorder’ also scoring above threshold on the AQ. (PNG 54 kb)

## Abbreviations

3D: Three-dimensional
ADC: Adult Developmental Coordination Disorder Checklist
AQ: Autism Spectrum Quotient
ASA: Autostereogram Self-Assessment
ASD: Autism spectrum disorder
CFA: Confirmatory factor analysis
CFI: Comparative Fit Index
DCD: Developmental coordination disorder
EFA: Exploratory factor analysis
MCAR: Missing completely at random
RMSEA: Root mean square error of approximation
SEM: Structural equation modelling
SRMR: Standardised root mean square residual
SSI: Stereopsis Screening Inventory
TLI: Tucker-Lewis Index
