# Supplementary material for: Does stereopsis account for the link between motor and social skills in adults?
Source: Mol Autism. 2018 Oct 24;9:55. doi: 10.1186/s13229-018-0234-4 (PMC6201514; doi:10.1186/s13229-018-0234-4)
Supplement: Supplementary file 1 — Table S1. A detailed breakdown of self-reported occupation, including faculty for those in education (where available) and sector for those in employment. (DOCX 25 kb) [file 13229_2018_234_MOESM1_ESM.docx]

*Table S1. A detailed breakdown of self-reported occupation, including faculty for those in education (where available) and sector for those in employment.*

| In education |  | Number of participants |
| --- | --- | --- |
|  | Secondary education | 7 |
|  | Tertiary education: arts | 24 |
|  | Tertiary education: science | 239 |
|  | Tertiary education: medicine and allied health professional | 10 |
|  | Tertiary education: teaching and education | 1 |
|  | Tertiary education: law | 4 |
|  | Level/faculty unknown | 30 |
| In employment |  |  |
|  | Accountancy, banking and finance | 5 |
|  | Business, consulting and management. | 15 |
|  | Charity and voluntary work. | 1 |
|  | Creative arts and design. | 25 |
|  | Energy and utilities. | 1 |
|  | Engineering and manufacturing. | 11 |
|  | Environment and agriculture. | 1 |
|  | Healthcare. | 19 |
|  | Hospitality and events management | 2 |
|  | Information technology | 42 |
|  | Law | 4 |
|  | Law enforcement and security | 2 |
|  | Leisure, sport and tourism | 1 |
|  | Marketing, advertising and PR | 2 |
|  | Military | 4 |
|  | Media and internet | 3 |
|  | Property and construction | 3 |
|  | Public services and administration | 18 |
|  | Research and academia | 21 |
|  | Retail | 10 |
|  | Sales | 1 |
|  | Science and pharmaceuticals | 9 |
|  | Social care | 4 |
|  | Teacher training and education | 15 |
|  | Transport and logistics | 2 |
| Not working or in education |  |  |
|  | Home maker | 10 |
|  | Retired | 7 |
|  | Unemployed | 19 |
