# Supplementary material for: Does stereopsis account for the link between motor and social skills in adults?
Source: Mol Autism. 2018 Oct 24;9:55. doi: 10.1186/s13229-018-0234-4 (PMC6201514; doi:10.1186/s13229-018-0234-4)
Supplement: Supplementary file 2 — Table S2. The psychiatric or organic illnesses self-disclosed in the feedback section of the questionnaire. Note that diagnoses were not collected routinely as part of the demographical data. The data below represent a number of co-morbidities: 32 diagnoses were disclosed by 24 participants (3.7% of the sample). (DOCX 24 kb) [file 13229_2018_234_MOESM2_ESM.docx]

*Table S2. The psychiatric or organic illnesses self-disclosed in the feedback section of the questionnaire. Note that diagnoses were not collected routinely as part of the demographical data. The data below represent a number of co-morbidities: 32 diagnoses were disclosed by 24 participants (3.7% of the sample).*

| Neuropsychiatric disorder |  | Number of participants |
| --- | --- | --- |
|  | Anxiety | 3 |
|  | Attention-deficit hyperactivity disorder | 3 |
|  | Autism spectrum disorder | 6 |
|  | Depression | 2 |
|  | Dyslexia | 1 |
|  | Pragmatic language impairment | 1 |
|  | Tourette’s syndrome | 1 |
| Ophthalmic diseases |  |  |
|  | Amblyopia | 1 |
|  | Poor vision | 8 |
|  | Strabismus | 5 |
| Hearing impairment |  |  |
|  | Deafness | 1 |
