# Supplementary figures and images for: Does stereopsis account for the link between motor and social skills in adults?
Source: Mol Autism. 2018 Oct 24;9:55. doi: 10.1186/s13229-018-0234-4 (PMC6201514; doi:10.1186/s13229-018-0234-4)

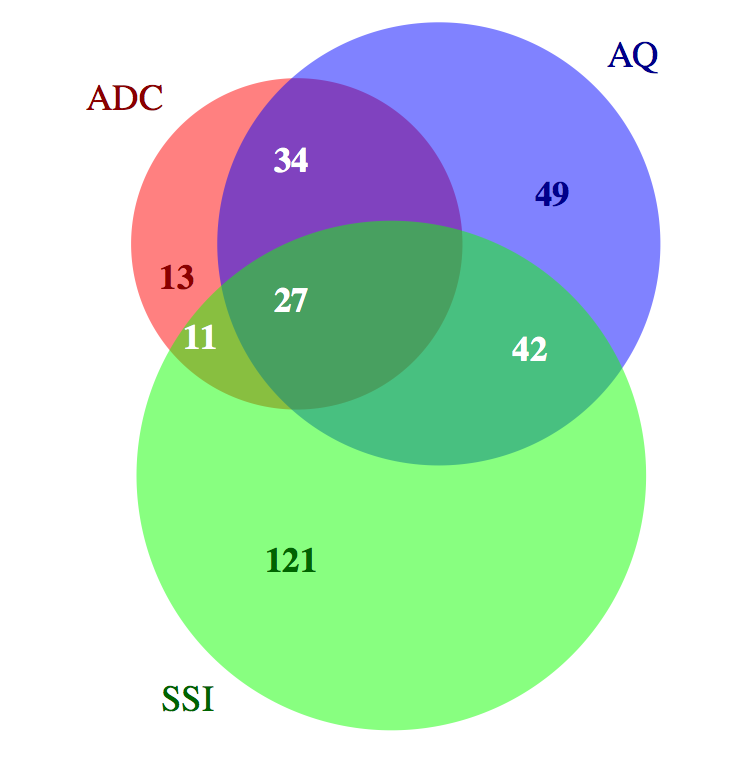

Supplement: Supplementary file 3 — Figure S1. Depicted is a 3-set Venn diagram where the size of the ovals indicates relative magnitude and the numbers within portray the number of participants who scored above threshold on th(at|ose) measure(s). Note that for the SSI, the higher threshold boundary indicating major stereopsis deficit was used. Descriptive statistics for questionnaire responses: It was not uncommon for participants who had a score above threshold for one measure to also score above threshold for at least one of the other measures. The most substantial amount of overlap between measures was for the AQ and the SSI, with 10.615% of the total participant sample scoring above threshold on both of these measures (note that the higher SSI threshold indicating major stereopsis deficit was used in this case). However, the largest degree of overlap was between the ADC and AQ, with 71.765% of participants who met the threshold for ‘probable developmental coordination disorder’ also scoring above threshold on the AQ. (PNG 54 kb) [file 13229_2018_234_MOESM3_ESM.png]
